# Clinical-dosimetric analysis of measures of dysphagia including gastrostomy-tube dependence among head and neck cancer patients treated definitively by intensity-modulated radiotherapy with concurrent chemotherapy

**DOI:** 10.1186/1748-717X-4-52

**Published:** 2009-11-12

**Authors:** Baoqing Li, Dan Li, Derick H Lau, D Gregory Farwell, Quang Luu, David M Rocke, Kathleen Newman, Jean Courquin, James A Purdy, Allen M Chen

**Affiliations:** 1 Departments of Radiation Oncology, University of California Davis Cancer Center, Sacramento, CA 95817, USA; 2 Departments of Applied Science, University of California Davis Cancer Center, Sacramento, CA 95817, USA; 3 Departments of Medical Oncology, University of California Davis Cancer Center, Sacramento, CA 95817, USA; 4 Departments of Otolaryngology-Head and Neck Surgery, University of California Davis Cancer Center, Sacramento, CA 95817, USA; 5 Departments of Public Health Sciences, University of California Davis Cancer Center, Sacramento, CA 95817, USA

## Abstract

**Purpose:**

To investigate the association between dose to various anatomical structures and dysphagia among patients with head and neck cancer treated by definitive intensity-modulated radiotherapy (IMRT) and concurrent chemotherapy.

**Methods and materials:**

Thirty-nine patients with squamous cancer of the head and neck were treated by definitive concurrent chemotherapy and IMRT to a median dose of 70 Gy (range, 68 to 72). In each patient, a gastrostomy tube (GT) was prophylacticly placed prior to starting treatment. Prolonged GT dependence was defined as exceeding the median GT duration of 192 days. Dysphagia was scored using standardized quality-of-life instruments. Dose-volume histogram (DVH) data incorporating the superior/middle pharyngeal constrictors (SMPC), inferior pharyngeal constrictor (IPC), cricoid pharyngeal inlet (CPI), and cervical esophagus (CE) were analyzed in relation to prolonged GT dependence, dysphagia, and weight loss.

**Results:**

At 3 months and 6 months after treatment, 87% and 44% of patients, respectively, were GT dependent. Spearman's ρ analysis identified statistical correlations (p < 0.05) between prolonged GT dependence or high grade dysphagia with IPC V65, IPC V60, IPC Dmean, and CPI Dmax. Logistic regression model showed that IPC V65 > 30%, IPC V60 > 60%, IPC Dmean > 60 Gy, and CPI Dmax > 62 Gy predicted for greater than 50% probability of prolonged GT dependence.

**Conclusion:**

Our analysis suggests that adhering to the following parameters may decrease the risk of prolonged GT dependence and dysphagia: IPC V65 < 15%, IPC V60 < 40%, IPC Dmean < 55 Gy, and CPI Dmax < 60 Gy.

## Introduction

Concurrent chemoradiation therapy using intensity-modulated radiotherapy (IMRT) has gained widespread acceptance as a definitive treatment for locally advanced head and neck cancer due to significant improvement in tumor control and organ preservation with the addition of chemotherapy, and promising advantage of increasing therapeutic gain using IMRT technique [1-4]. However, it is becoming increasingly clear that chemoradiation strategy is associated with an increased incidence and severity of swallowing-related toxicities, including high-grade dysphagia, severe weight loss, and prolonged dependence on gastrostomy tube (GT) for fluid and nutritional support [5-7].

Indwelling GT has been shown to compromise quality of life because it may cause infection and physical discomfort, distort patient's self-esteem, and induce anxiety, depression, and social isolation [8]. Presently there is a lack of data associating GT dependence and dosimetric parameters among patients undergoing definitive chemoradiotherapy using IMRT for head and neck cancer [9,10]. This is of practical significance since, as a result of IMRT optimization, radiation doses can potentially be "dumped" to unspecified anatomical areas including those related to dysphagia that have not yet been rigorously investigated [11]. In a prospective trial using IMRT, Feng et al demonstrated the importance of monitoring dose to the pharyngeal constrictor muscles, the cervical esophagus (CE), and the glottic and supraglottic larynx (GSL) [12]. The purpose of the present study was to investigate the potential association between radiation dose to these structures vital for swallowing and severity of dysphagia, notably prolonged GT dependence, among a cohort of patients undergoing definitive IMRT chemoradiation for locally advanced head and neck cancer.

## Methods and materials

### Patient characteristics

This was a retrospective study approved by the Institutional Review Board at the University of California, Davis (UCD). Between January 2003 and January 2007, forty-eight patients with newly diagnosed squamous cell carcinoma involving the oral cavity, oropharynx, larynx or hypopharynx were treated with definitive chemoradiation consisting of IMRT and cisplatin at the UCD Cancer Center. Seven patients who either developed locoregional recurrence or were lost during follow up were excluded from the study. Two patients who refused prophylactic placement of a GT were also excluded. The remaining 39 patients included in the study. The median follow up was 15.6 months (range, 4.5 to 52 months), with 27 patients followed greater than 1 year. All patients received prophylactic placement of a GT prior to starting treatment. The GT was subsequently removed upon resolution of high grade dysphagia and stabilization of weight after treatment. Physician judgment if GT needed to be maintained was based on the criteria that 1) the patient's weight could not be maintained with less than two cans of supplemental feeding per day, or 2) the patient could not tolerate solid food without complaints of dysphagia, odynophagia or aspiration. None of the patients required GT reinsertion once the GT was initially removed after completion of radiation therapy. Table 1 shows patient characteristics of the study population.

**Table 1:** Patient and tumor characteristics.

*Variable*	No. patients	%
***Age***	Mean 56,	
Continuous	range 32-77	
***Gender***		
Male	32	82
Female	7	18
Active smoking*		
Yes	30	77
No	9	13
***Alcohol use***		
Heavy**	11	28
Others	28	72
***KPS***		
80-100	25	64
60-70	14	36
***Primary site***		
Oral cavity	2	5
Oropharynx	25	64
Larynx	6	15
Hypopharynx	3	8
Unknown primary	3	8
***T stage***		
T0, 1, 2	25	64
T3, 4	14	36
***N stage***		
N0/N1	16	41
N2	19	49
N3	4	10
***Chemo regimen***		
CDDP-based	33	85
Others	6	15
***Post RT neck dissection***		
Yes	5	13%
No	34	87%

*: currently smoking or smoking history within one year.

**: self reported active heavy alcohol drinking or more than one 6-pack of beers per day.

### Target volume delineation

The gross tumor volume (GTV) was specified as the gross extent of tumor as demonstrated by preoperative imaging and physical examination including endoscopy. Grossly positive lymph nodes were defined as any lymph nodes greater than 1 cm or those with a necrotic center. The high-risk clinical target volume (CTV1) was defined as the GTV plus a margin of 1-2 cm to account for microscopic disease spread. The CTV2 generally included the prophylactically treated cervical and supraclavicular neck. A CTV3 was also created to designate an area at lowest risk within the prophylactically treated low neck. The low neck was encompassed within the IMRT plan in all cases, and thus a separate anterior low-neck field was not used. Depending on disease site, the planning target volume (PTV) contained an automated 0.5 cm expansion of the CTV surfaces to account for patient setup error to create PTV1, PTV2, and PTV3, if necessary. The tumor volumes and sensitive normal structures were delineated on serial treatment planning CT images. Structures considered to be critically at risk included the spinal cord, optic nerves, optic chiasm, orbits, lens, brainstem, and parotid glands. No overlap between CTVs and uninvolved critical adjacent tissues was permitted for optimization purposes.

### Dose specification

For patients receiving definitive radiation therapy, treatment plans were designed to provide a dose of 68 to 72 Gy (median, 70 Gy) to 95% or greater of the PTV1 while sparing neighboring critical structures. The prescribed dose to PTV3 was 54 to 56 Gy. Dose to PTV2 ranged from 59.4 to 63 Gy (median, 60 Gy). For critical normal structures, dose constraints were designed to limit the maximum dose, whenever possible, to 1% of the volume to 54 Gy for the brainstem and optic nerves, 45 Gy for the spinal cord and optic chiasm, 60 Gy for the temporal lobes, and 30 Gy to 50% of the contralateral parotid gland. Treatment was by continuous-course IMRT with once-a-day treatment. Because our goal was to prescribe 1.8 Gy per fraction to the PTV2 daily, the PTV1 received a higher dose per fraction, typically 2.0 Gy or 2.12 Gy per fraction, and PTV3 typically 1.6-1.7 Gy per fraction.

### Delineation of swallowing structures

**Figure 1:**
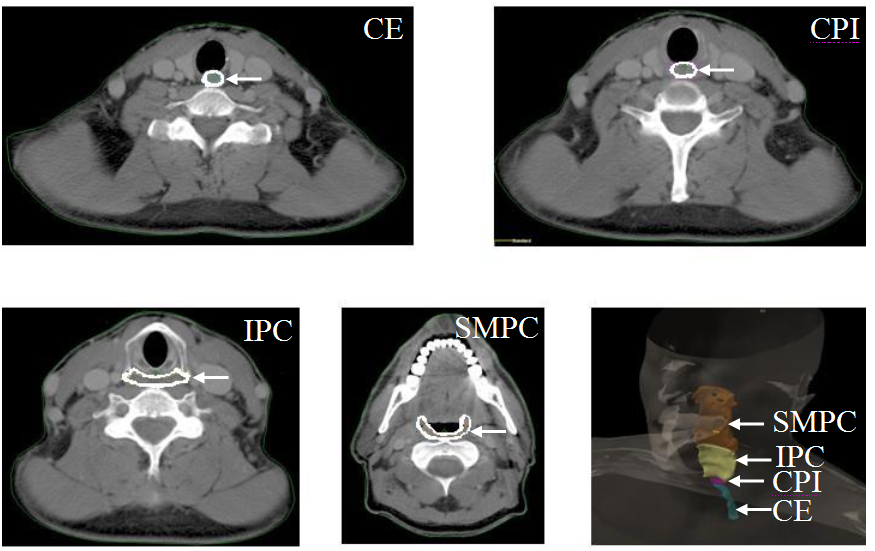
**Delineation of the swallowing structures on axial slices from simulation CT and 3D reconstructed image**. SMPC = superior and middle pharyngeal constrictor; IPC = inferior pharyngeal constrictors; CPI = cricoid pharyngeal inlet; and CE = cervical esophagus.

The IMRT treatment plans of all 39 patients treated by definitive chemoradiation were retrieved from archival records. With the help of a board-certified head and neck surgeon, the swallowing structures were contoured on axial CT slides as previously described [11-14] (Fig. 1). Briefly, the pharyngeal constrictor (PC) was outlined as a single structure for which the cranial-most extent was the caudal tips of the pterygoid plates and the caudal-most extent was the inferior border of the cricoid cartilage. For purposes of analysis, the constrictors were considered as one structure and were also schematically divided into two parts: the superior and middle PC (SMPC) was defined from the caudal tips of the pterygoid plates through the lower edge of the hyoid, at the level of C2, C3 and upper C4. The inferior PC (IPC) was defined from below the hyoid through the inferior edge of the cricoid, with attachment to the inferior horn of thyroid cartilage, at the level of lower C4, C5 and upper C6. On non-contrast CT images, IPC can be identified as a structure with faint enhancement of mucosa surrounded by a thin intramural fat plate which facilitates the exclusion of the posterior cricoarytenoid muscle. The cricopharyngeal inlet (CPI) was defined as an oval structure of 1 cm in length, with lack of intramural fat plate. It extends from the caudal cricoid to the first tracheal ring, and is located at the level of lower C6. The CE was contoured as a round structure, caudal to the CPI, with its caudal-most extent corresponding to the thoracic inlet. With the above structures delineated on the axial CT slices, tabular differential dose-volume histogram (DVH) data for all the structures were re-computed, taking into consideration the dose actually delivered.

### Chemotherapy regimens

The majority (85%) of the patients received bolus cisplatin (100 mg/m^2^) given every 3 weeks on days 1 and 22. The remaining patients received either weekly carboplatin (AUC = 2) or weekly paclitaxel (50 mg/m^2^) for 6 weeks. Cetuximab was not used among any of the patients in the study. None of the patients received sequential induction or consolidation therapy.

### GT management

The GT was inserted by the Department of Interventional Radiology at UCD, and was changed every three months. In 3 patients (8%), additional GT changes were performed due to complications such as infection or obstruction. Patients were encouraged to undergo feeding by mouth for as long as it was tolerable. Body weight and toxicity (dysphagia, xerostomia, mucositis, nausea, vomiting, constipation, diarrhea, dysguesia, difficulties chewing) were assessed and addressed with patients weekly. We used the American Dietetic Association Medical Nutrition Therapy (MNT) Protocol for Cancer (Radiation Oncology) and the UCD Enteral Nutrition Guidelines. Decisions to wean off enteral feeds were based on individual patient and chemoradiation-induced toxicities with specific emphasis placed on inability to consume adequate oral nutrition and fluid, dysphagia, and prevent uncontrolled involuntary weight loss. Patients were weaned off enteral nutrition support when 1) the patient's weight could be maintained with less than two cans of supplemental feed per day, and 2) the patient could have certain solid food without complaints of dysphagia, odynophagia or aspiration.

### Follow-up evaluation

Patients were typically seen 2 to 3 weeks after completion of radiation therapy and then every 3 months thereafter for the first year, every 6 months for the second and third year, and then annually. The mean follow up time was 16.2 months (range 4.5-52 months). If a persistent neck node was found on physical examination after completion of IMRT and/or was positive on PET/CT at 2 months follow-up, salvage neck dissection was performed. Duration of GT was defined as the interval between RT completion and the date of its removal, or until the date of last follow-up or death if the GT was still present. Prolonged GT dependence was defined as GT more than the median GT duration. Two patients whose follow-up time were less than the mean GT duration were excluded from further analysis of clinical-dosimetric association. Body weight and patient-reported dysphagia were recorded during each follow up. The lowest body weight during the follow up period was used. Severe weight loss was defined as more than 15% weight loss. Patient-reported dysphagia was assessed with the validated UWQOL questionnaires given to patients during each follow-up visit. It contained one swallowing question with five possible answers ("I swallow normally", (grade 0); "I cannot swallow certain solid food", (grade 1); "I can only swallow soft food", (grade 2); "I can only swallow liquid food", (grade 3); and "I cannot swallow", (grade 4). Grade 3 and grade 4 defined high grade dysphagia. Mucositis and xerostomia was evaluated weekly during treatment, and at follow-up, based on Common Terminology Criteria for Advanced Events (CTCAE), version 2.0. Accordingly, high grade mucositis was defined as confluent pseudomembranous reaction with continuous patches > 1.5 cm (grade 3) or necrosis or deep ulceration; this may include bleeding not induced by minor trauma or abrasion (grade 4).

### Transnasal esophagoscopy (TNE), flexible endoscopic evaluation of swallow (FEES), and aspiration pneumonia work up

Patients with grade 2 or greater dysphagia beyond 3 months after radiation were referred for TNE. The TNE technique has previously been described [15,16]. At the discretion of the physician, a FEES or aspiration pneumonia work up (bacterial culture and chest X-ray) was performed. FEES allows direct visual assessment of many swallowing functions including muscular function, premature spillage, pooling, laryngeal penetration, and presence of aspiration. In brief, the patients were examined seated upright without anesthesia. Liquid (colored water), pureed food (yogurt), and chewable food (bread) were ingested while the hypopharynx and laryngeal contents were viewed with the fiberscope. The results were scored as "little", "moderate", or "severe" using the following variables: residue, penetration, and aspiration of three different types of diet (water, yogurt, and bread), and mucus stases. Aspiration pneumonia was defined as cultured bacterial pneumonia with radiographic evidence of infiltration.

### Statistical analysis

Data analysis and graphs were completed using the R software program (R Development Core Team, 2006; R Foundation for Statistical Computing, Vienna, Austria). Spearman's ρ and univariate regression were used to calculate the correlation of each of these identified DVH parameters and individual dependent binary variable (absence or presence of prolonged GT days, grade 3+ dysphagia, and severe weight loss). A logistic model, p = 1/{1+exp [-(α +β *dose or volume of structure)]}, was used to calculate the probability of developing prolonged GT days, grade 3+ dysphagia, or severe weight loss. The unknown parameters α and β were estimated with the maximum likelihood method. A test was also performed whether the hypothesis β = 0 can be rejected. A p value of < 0.05 was interpreted as being statistically significant from zero. Confidence intervals (95%) were determined. Multivariate regression was not used due to the model instability caused by co-linearity between DVH parameters (V40, V50, V60, V65, Dmax, Dmean). Wilcoxon rank-sum analysis was preformed to identify DVH parameters that statistically correlated with esophageal stricture.

## Results

### Swallowing outcomes after treatment

At 3 months and 6 months after treatment, 87% and 44% of patients, respectively, were GT dependent (Table 2). The results of physician-assessed high grade dysphagia were consistent with that of GT dependence, given that majority of high grade dysphagia patients were grade 3 with GT dependence. Due to data redundancy, results of observer-assessed high grade dysphagia were not reported. Using the UWQOL instrument, 17 patients (44%) reported high grade dysphagia at any point during or after treatment. The median percent of maximum weight loss was 12% (range, -4% to 21%).

**Table 2:** Toxicity after treatment

	*3 month (%)*	*6 month (%)*
***GT dependence***	87%	44%

***Self-reported dysphagia grade ≥ 3***	33%	21%

***Mucositis*** ***grade ≥ 3***	23%	5%

***Xerostomia grade ≥ 2***	43%	36%

*Abbreviation*: GT = gastrostomy tube

More than half (54%) of the patients had Grade 3 or 4 mucositis at some point after radiation, with 23% and 5% having severe mucositis at 3-month and 6-month follow up evaluation respectively (Table 2). However, GT dependence did not improve as rapidly and still persisted in 87% and 44% of patients at 3 months and 6 months, respectively. There was a lack of temporal association between high grade mucositis and prolonged GT dependence of more than 192 days on statistical analysis (p > 0.05). Grade 2 or higher xerostomia was found in 43% and 36% at 3-month and 6-month follow up evaluation respectively, and persisted in 31% at the last follow-up (Table 2).

Twelve of the patients with high grade dysphagia underwent TNE. Five of them developed stricture at the upper esophageal sphincter at the level of the cricopharyngeus muscle, including one with complete luminal stenosis. All of them underwent dilatation at the time of TNE to relieve any physical obstruction. Four out of the seven patients who underwent FEES had finding of moderate or severe aspiration to one of the diets. Eight patients had aspiration pneumonia work up, and only one of them was diagnosed.

The clinical factors listed in Table 1 were included in both univariate and multivariate analysis of prolonged GT dependence. Smoking (active smoking or smoking history within one year) was identified as the only significant factor predictive for prolonged GT dependence (p = 0.03). Other clinical factors, including age, gender, history of alcohol use, KPS, tumor site, T stage, N stage, and type of chemotherapy regime are not associated with prolonged GT dependence. Similar analysis of high grade dysphagia revealed active smoking (p = 0.03) and T stage (p = 0.04) as significant factors. No other predisposing parameter was found to be statistically significant. In terms of severe weight loss, no predisposing parameter was identified to be statistically significant (data not shown). A total of 5 patients underwent post-treatment neck dissection. Both univariate and multivariate analysis did not reveal neck dissection as a significant factor for prolonged GT dependence, high grade dysphagia, or severe weight loss.

### DVH analysis for prolonged GT dependence

The DVH parameters for all the swallowing structures (SMPC, IPC, CPI, and CE) were listed in Table 3. Significant factors (p < 0.05) for prolonged GT dependence were revealed using Spearman's ρ test and subsequent univariate logistic regression in an attempt to identify dose-volume effect for GT duration longer than 192 days versus less than 192 days. These factors are IPC V65 (p = 0.003), IPC V60 (p = 0.002), IPC V50 (p = 0.042), IPC Dmean (p = 0.016), and CPI Dmax (p = 0.011). CPI V60 has p value of 0.050. DVH analysis was also performed on a combined structure (IPC, CPI and CE). No statistically significant factor was identified (p > 0.05).

**Table 3:** Swallowing structure DVH parameters (median value and range) and p values for association with GT dependence

	V40 (%)	V50 (%)	V60 (%)	V65 (%)	Dmax (Gy)	Dmean (Gy)
***CE***						
*GT > 192 d*	57 (1-86)	24 (0-79)	0 (0-33)	0 (0-1)	61 (45-77)	36 (20-53)
*GT ≤ 192 d*	65 (17-98)	27 (0-86)	0 (0-13)	0 (0-0)	57 (44-76)	36 (21-56)
	p = 2.671	p = 0.524	p = 0.173	P = 0.169	p = 0.238	p = 0.383
***CPI***						
*GT > 192 d*	100 (60-100)	100 (4-100)	8 (0-100)	0 (0-100)	64 (56-78)	57 (42-69)
*GT ≤ 192 d*	100 (93-100)	95 (3-100)	0 (0-73)	0 (0-27)	58 (53-67)	53 (43-62)
	p = 0.512	p = 0.069	p = 0.050	P = 0.062	p = 0.011	p = 0.083
***IPC***						
*GT > 192 d*	100 (97-100)	100 (76-100)	81 (40-100)	42 (21-100)	74 (66-79)	64 (54-70)
*GT ≤ 192 d*	100 (95-100)	92 (40-100)	37 (10-84)	15 (0-60)	72 (68-77)	55 (46-68)
	p = 0.367	p = 0.042	p = 0.002	P = 0.003	p = 0.057	p = 0.016
***SMPC***						
*GT > 192 d*	100 (60-100)	100 (56-100)	94 (45-100)	72 (21-93)	76 (66-79)	67 (38-72)
*GT ≤ 192 d*	100 (84-100)	97 (70-100)	90 (25-100)	60 (0-95)	76 (70-79)	65 (50-71)
	p = 0.378	p = 0.072	p = 0.063	P = 0.091	p = 0.252	p = 0.086

*Abbreviation*: DVH = Dose-volume histogram; GT = gastrostomy tube; SMPC = superior and middle pharyngeal constrictor; IPC = inferior pharyngeal constrictors; CPI = cricoid pharyngeal inlet; and CE = cervical esophagus; Dmax = maximum dose; Dmean = mean dose.

**Figure 2:**
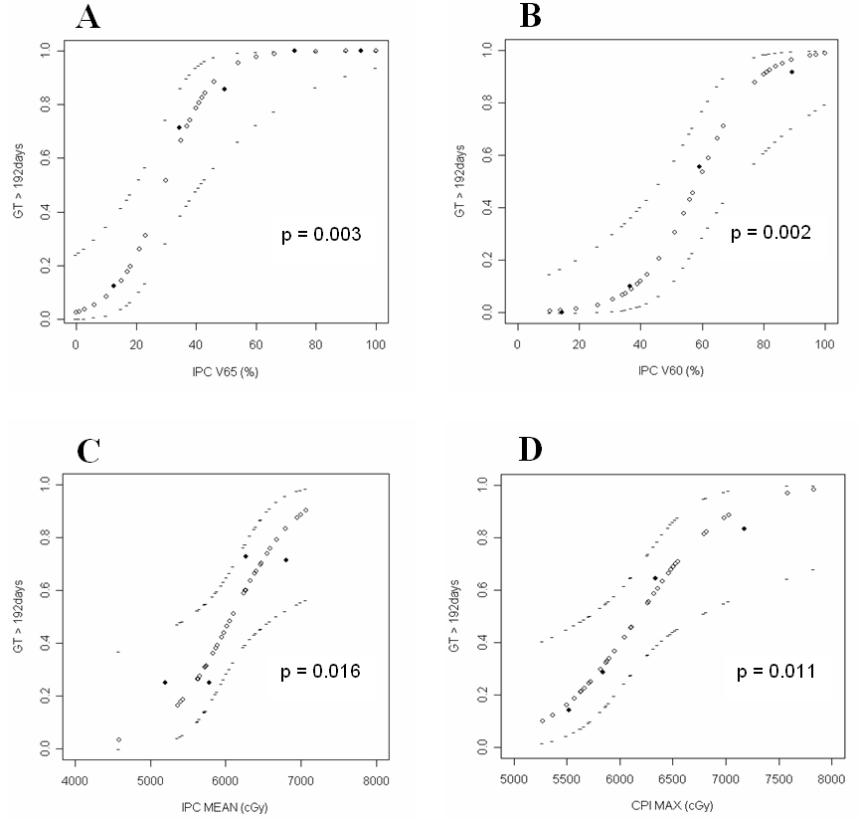
**Volume-response or dose-response relationship for the average probability of having prolonged GT dependence and the volume of the IPC receiving more than 6500 cGy (A), or 6000 cGy (B), or the mean dose to the IPC (C), or maximum dose to CPI (D)**. GT = gastrostomy tube; IPC = inferior pharyngeal constrictors; CPI = cricoid pharyngeal inlet. The ◇ lines plot the mean risk; the - lines plot the estimated upper and lower limits of 95% confidence interval. The ♦ points depict the observed values.

The results of dose-response relationships and volume-response relationships for prolonged GT dependence are presented in Fig. 2. IPC V65 more than 30%, IPC V60 more than 60%, IPC Dmean more than 60 Gy, and CPI Dmax more than 62 Gy predicted for a greater than 50% probability of developing prolonged GT dependence. For IPC V50, the dose/volume-response relationships results were not clinically meaningful.

In view of the strong dosimetric-clinical correlations for CPI and IPC, we repeated the above analysis with exclusion of three patients whose primary disease overlapped with the relevant structures (CPI and IPC). The same DVH parameters were observed as significant factors for prolonged GT dependence.

### DVH analysis for high grade dysphagia, severe weight loss, and stricture

Spearman's ρ test and subsequent univariate logistic regression analysis revealed significant associations between several dosimetric parameters and grade 3+ patient-reported dysphagia. These factors are IPC V65 (p = 0.040), CPI Dmax (p = 0.037), and CPI V60 (p = 0.046). Further analysis of dose-response relationships and volume-response relationships revealed that IPC V65 more than 65%, CPI V60 more than 78%, CPI Dmax more than 70 Gy were associated with more than 50% probability of developing high grade dysphagia. Similar analysis did not reveal statistically significant DVH predictors for severe weight loss (data not shown). Wilcoxon rank-sum analysis revealed significant associations between stricture and two dosimetric parameters (CPI V65, CPI Dmax).

## Discussion

It has been a common observation that a correlation exists between dysphagia and radiation doses to the anatomic structures responsible for swallowing in patients undergoing definitive chemoradiation for head and neck cancer. However, the present study is the first to document a relationship between various dosimetric parameters and prolonged GT dependence. Notably, we were able to identify DVH parameters which were significantly associated with prolonged GT dependence, including V65 of the IPC, V60 of the IPC, mean dose to the IPC, and maximum dose to the CPI. Based on these dose/volume-response relationships, we currently recommend IPC V65 less than 15%, IPC V60 less than 40%, IPC Dmean less than 55 Gy, and CPI Dmax less than 60 Gy as potentially important DVH constraints to guide IMRT planning in an attempt to significantly reduce the risk of swallowing dysfunction and prolonged GT dependence.

Our findings demonstrate the importance of IPC and CPI dosimetric parameters for developing swallowing dysfunction and are consistent with those from several recently published studies. Caglar et al showed that a mean dose to the IPC of more than 54 Gy and IPC V50 of more than 50% were the most significant predictors for aspiration or stricture development [17]. Levendag et al identified dose-response relationship between dysphagia for solids (p < 0.02) or aspiration episodes (p < 0.02) and mean dose to IPC. A mean dose of 33 Gy to IPC was estimated as the threshold for 20% risk of dysphagia for solids [14]. Furthermore, Dornfeld et al reported that a more restrictive diet one year after treatment is significantly correlated with higher average dose delivered to the constrictor muscles (lateral pharygeal wall) at the level of false vocal cord [18]. Jensen et al demonstrated that dose above 60 Gy to the upper esophageal sphincter could result in higher risk of late swallowing dysfunction [19]. This well documented association between high dose to IPC or CPI and prolonged GT dependence was also supported by two earlier reports showing that patients were more likely to have prolonged GT dependence and high grade dysphagia when treated with extended-field IMRT rather than being treated with an upper IMRT fields junctioned with an anterior neck field. This is thought to be due to the presence of midline block in an anterior neck field to prevent unanticipated high dose radiation to structures including larynx, IPC, CPI and CE [20,21].

The significant dose-volume effect relationships regarding prolonged GT dependence for IPC and CPI could be explained by the role of the upper esophageal sphincter (UES) in the normal swallowing process. The UES is a functional entity that is composed of three muscles: the IPC muscle, the CPI muscle, and the upper esophageal muscle. The UES opens by relaxation of the three closing muscles, traction by IPC and other muscles that attached to the hyoid bone and thyroid cartilage, anterior movement of the larynx, and pulsion of the bolus. The various muscles of the UES behave differently during its many dynamic states, so that similar functions are accomplished by different muscles. Any impairment of the CPI and IPC could result in dysphagia. In addition, UES is considered a high pressure zone, with the highest pressure at the region around IPC where proprioceptive units were identified. A cause of dysphagia could also be attributed in part to the failure of sensation and timely response to the bolus passing through this region. The importance of IPC and CPI is validated by our finding that patient-reported dysphagia was highly correlated with the dose to the two structures.

In addition to the IPC and CPI, several other anatomic structures have been reported as dysphagia/aspiration related with significant dose-volume relationship. These structures include GSL and PC, with superior PC having the strongest dose-response association [11,12]. The importance of superior and middle PC for swallowing after radiation therapy was also shown by Teguh et al [14,22,23]. Although our study failed to find a significant a correlation between GT dependence and dose to the GSL, SMPC, or PC as a whole, this could potentially be explained by the differences in patient characteristics. In the above mentioned studies, only oropharynx and nasopharynx patients were included. As such, our results are consistent with those from Caglar et al that the mean dose or V50 to IPC, not the superior PC, were significant predictors for aspiration or stricture development [17].

Prolonged GT dependence is regarded by most head and neck cancer patients as contributing to compromised quality of life because it may cause infection and physical discomfort, distort patient's self-esteem, and induce anxiety, depression and social isolation [8]. This is of increasing concern in recent years when concurrent chemoradiation for tumor control and organ preservation has gained widespread practice but is associated with high rate of severe late dysphagia, including prolonged GT dependence [5]. Multiple large randomized trials testing intensified chemoradiation regimens reported GT rates of about 70%, and chronic tube dependence of 10-20% [3,6,7]. In a recent study where 95% of the chemoradiation patients had prophylactic feeding tubes placed before treatment, Caglar et al reported prolonged GT dependence in 37% of the patients, with a median GT duration of 112 days after radiation completion [17]. Notably, we also identified smoking as a risk factor for GT dependence. The etiology for smoking induced dysphagia is likely multifactorial and related to prolonged tissue recovery secondary to nicotine induced hypoxia, the appetite reducing effects of nicotine, or mucosal irritation. Multiple previous works have similarly associated smoking with higher rates of toxicity including aspiration and esophageal stricture after radiation therapy [24,25].

Notably, 5 out of the 12 (42%) patients with high grade dysphagia developed upper esophageal stricture in this study. This high incidence could have resulted from detection bias, small patient number, or most likely, patient over-reliance on a GT which led to less swallowing and allowing scar and stricture formation. The last possibility is supported by results from Caudell et al who demonstrated a trend toward an association (p = 0.09) between GT dependence and pharyngeal stricture or stenosis [26]. Another explanation for this high incidence of stricture among patients with high grade dysphagia could be its relatation with CPI Dmax, which was significantly associated with both high grade dysphagia and stricture formation. In spite of this high incidence among patients with high grade dysphagia, the overall incidence of stricture in all patients is 12% (5/41) in our study, consistent with stricture rate of 17%-37% in other studies [17,26-28].

It is important to note that this study was retrospective with inherent limitations. First, the lack of systematic evaluation of some of the major end points of late dysphagia using TNE or FEES prevented more robust analysis using more objective endpoints. Another limitation of the study was the relatively short follow-up with a median duration of 15.6 months. However, most of the endpoint events occurred less than 1 year after treatment. Given the small number of events, we conjecture that a sub-analysis of swallowing function assessed at greater than 1 year after treatment would likely not change our findings. Nonetheless, we do acknowledge that further studies with more comprehensive objective endpoints with prolonged follow-up may be necessary to yield a more thorough evaluation. This is well exemplified in a recently published retrospective study of patients with more than 1 year follow-up, where a composite of 3 objective endpoints (GT dependence, aspiration, and pharyngoesophageal stricture) were successfully used as surrogates for severe long-term dysphagia [26].

It must also be recognized that the majority of patients in the present study presented with oropharynx cancer, and this fact may have biased our findings. Teguh et al, for instance, demonstrated that patients with base of tongue disease experienced more severe dysphagia than those with tumors at other sites [22]. In addition to oropharynx primary, the larynx, hypopharynx and pharyngeal wall were also found to predispose to dysphagia more so than other regions of the head and neck [26-29]. In contrast, however, Logemann et al showed no differences in the frequency of dysphagia across different head and neck disease sites [30], which is further supported by a large prospective study that excluded disease site as a statistically significant factor for quality of life changes among head and neck cancer treated with radiation therapy [31].

Lastly, we were unable to control for potentially confounding factors which may have also predisposed to swallowing dysfunction including severe mucositis, pre-treatment dysphagia and post-treatment xerostomia. We acknowledge that the endpoints in this study, such as high grade dysphagia and prolonged GT dependence may have been confounded by the development of severe acute mucositis or its consequential late effects, such as submucosal edema, fibrosis, scarring, soft tissue necrosis, impaired sensory or motor function, and loss of mucosal compliance. This is based on the rationale that high dose radiation to a large volume of the constrictor muscles (resulting in high values of V60 and V65) also results in high dose to large volumes of mucosal surface which is believed to lead to more severe mucositis [32-34]. However, since mucositis tends to be self-limiting and an acute, rather than late side-effect of radiation therapy, we believe that the potential confounding effects are minimal. Furthermore, the present study demonstrated a lack of significant association between acute mucositis and prolonged GT dependence, which is consistent with the findings of a dissociation between acute mucositis and dysphagia by Mekhail et al [27] and it is supported by recent data from Anand et al showing no correlation between long-term dysphagia and acute mucositis (Grade 3, 4) in spite of the severe mucosities that developed in 53% of locally advanced head neck cancer patients treated with IMRT [35]. In addition, we were unable to completely exclude the possibility of other confounding factors such as pre-treatment dysphagia or post-treatment severe xerostomia as confounding factors. Multiple studies suggested that very few patients with newly diagnosed head and neck cancer have severe dysphagia or aspiration prior to definitive treatment [29,36,37]. Moreover, we could not completely rule out xerostomia as a confounding factor in spite of the reduction in the risk of this symptom associated with parotid gland sparing IMRT. Xerostomia secondary to chemotherapy or radiation therapy has been suggested to significantly affect food bolus formation and swallowing function, and contribute significantly to dysphagia after chemoradiation [38,39]. In particular, Teguh et al demonstrated a strong correlation between dry mouth and sticky saliva with dysphagia-related quality of life such as normalcy of diet and odynophagia [22]. As a result of these potential confounding factors, it remains difficult to definitively establish a cause-effect association in spite of the significant dose/volume-response relationship between some key DVH parameters and risk of prolonged GT dependence. A larger prospective study is required in the future to further investigate theses associations.

## Conclusion

We provided evidence that prolonged GT is correlated with DVH parameters for patients with locally advanced head and neck cancer treated with definitive concurrent chemotherapy and IMRT. To minimize the risk of prolonged GT dependence, we currently strive to keep IPC V65 less than 15%, IPC V60 less than 40%, and maintain IPC Dmean less than 55 Gy, and CPI Dmax less than 60 Gy during IMRT planning in an attempt to decrease the risk of prolonged GT dependence. It should be noted that these guidelines are implemented on a case-by-case basis considering such factors as tumor extent and location. Future directions include large-scale prospective trials aiming to assess the clinical benefits gained by applying these dosimetric strategies. Lastly, the proposed dosimetric constraints should not replace the effort of early swallowing therapy and exercises which resulted in maximal swallowing recovery in several studies [5,40].

## Competing interests

The authors declare that they have no competing interests.

## Authors' contributions

BL and AMC conceived of the study, and participated in its design, carried out data collection, data analysis, manuscript writing, and coordination. DL and DMR performed statistics analysis. DHL performed data collection regarding chemotherapy regimens and participated in manuscript writing. DGF and QL performed dysphagia data collection including TNE and FEES, and delineation of swallowing structures. KN and JC performed data collection regarding GT management. JAP performed physics consult on re-computation of DVH. All authors read and approved the final manuscript.
